# Efforts to Reach More Children with Effective Vaccines Through Routine Immunization in The WHO African Region: 2013-2015

**Published:** 2018-07-02

**Authors:** Blanche Anya, Joseph Okeibunor, Richard Mihigo, Alain Poy, Felicitas Zawaira

**Affiliations:** WHO Regional Office for Africa, Brazzaville, Congo

**Keywords:** Routine Immunization, Vaccines, African Region

## Abstract

**Background:**

Some progress has been made in expanding immunization in the African Region over the last four decades. However, an estimated 22% of the eligible children in the African Region, located in four countries of the African Region (Democratic Republic of the Congo, Ethiopia, Nigeria and South Africa), continue to miss vaccination services for various reasons. This paper documents the status of routine immunization in the African Region.

**Methods:**

Programme records, reports and statistics were reviewed for this paper.

**Results:**

Challenges remain in reaching an estimated 20–30% of children across the Region. In addition to the traditional vaccines (DTP, measles, polio and tuberculosis) newer ones, such as for Pneumococcal conjugate vaccine (PCV) and rotavirus, are being rolled out in the Region but uptake and coverage are slow and patchy both within and between countries.

**Conclusion:**

The new regional strategic plan for immunization 2014–2020 is intended to provide policy and programmatic guidance to Member States, in line with the 2011–2020 Global Vaccine Action Plan (GVAP), in order to optimize immunization services and assist countries to further strengthen their immunization programmes.

## Introduction

Vaccination remains one of the greatest public health accomplishments in protecting lives. It has been credited with significant reduction incidence of most vaccine-preventable diseases, therefore contributing to the reduction of morbidity and mortality due to vaccine preventable diseases^1^–^6^. Smallpox, a human and disease and rinderpest, which affect livestock, were eliminated through broad immunization with effective vaccines^7^. In the African Region, immunization programmes have recorded remarkable successes in delivering effective vaccines and ensuring equitable vaccination of the populations irrespective of social location, class, ethnicity or religion. Immunization coverage rates have shown upward trends over the years and access has increased remarkably even in previously hard to reach populations. Following global, regional and national efforts in the promotion of immunization, DTP3 coverage, the acknowledged indicator of the performance of immunization programmes, rose from 5% in 1980 to 76% in 2015 in the African Region^8^–^10^.

There has also been significant progress in the introduction of new and under-utilized vaccines in Africa^10^. Currently, many more children and adolescents receive vaccines to prevent diseases associated with childhood and adolescence, such as pneumonia, diarrhoea, yellow fever, measles and human papillomavirus (HPV). By 2012, 81% (27/33) of the countries at the risk for yellow fever had introduced the vaccination into their Expanded Programme on Immunization (EPI) schedules^11^, compared to 27% (9/33) in 2000^9^.

Pneumococcal conjugate vaccine (PCV) and rotavirus vaccines had been introduced in 37 and 29 of the 47 Member States as of the end of 2015, respectively. Meningitis A vaccine has been introduced since 2010 in campaigns in 16 countries of the meningitis belt in the African Region, with nearly 260,000,000 persons vaccinated since its introduction (WHO AFRO database 2015). Surveillance for diseases targeted by these new vaccines has been strengthened in all countries as part of monitoring disease trends and vaccine impact.

The strengthened disease surveillance has shown a decrease in the burden of vaccine-preventable diseases (VPD) as illustrated by the decline in the incidence of meningitis due to *Neisseria meningitidis* serotype A. As of December 2015 there has been no laboratory confirmed case of meningococcal A meningitis among vaccinated persons. Therefore, the Region is on course towards achieving the goal of eliminating meningococcal meningitis A outbreaks as a major public health problem.

Associated with immunization also is the eradication, elimination and control of VPDs in the Region. A clear case is the successful interruption of wild polioviruses (WPV) transmission in the Region^12^. With the use of effective vaccines, the last reported cases of the WPV types 2 and 3 were made in 1998 and 2012, respectively. Between 2000 and 2014, the number of African countries endemic with the WPV1 decreased from 12 to one. New WPV cases reported in 2012 decreased by 63%, with 128 cases in four countries (Nigeria, Cameroon, Guinea and Ethiopia) compared to 350 cases in 12 countries in 2011^13^. By July 2014, Nigeria, the only country endemic with the transmission of WPV in the Region, recorded its last WPV case and was removed from the list of endemic countries in 2015.

Similar successes have been recorded against other VPDs. This is the case for measles and maternal and neonatal tetanus elimination. As of December 2015, a total of 36/47 countries in the region had eliminated maternal and neonatal tetanus.

There has been 78% decline in measles case reports since 2000, from a reported number of 520,102 in the year 2000 to 113,938 in 2015. (African Regional Measles Elimination Status Report, August 2016, IVD, AFRO). However, there was a sharp increase in reported cases between the years 2010 – 2011, owing to the various outbreaks reported across the Region, especially in southern African countries. These outbreaks were mainly driven by a shift of epidemiological susceptibility to older age groups. The increase in the case reports noted in 2012 and 2013 represents large outbreaks documented in DR Congo and Nigeria, which contributed 88,381 and 52,852 cases respectively in 2013, amounting to 83% of the total 171,178 cases reported in that particular year. Between 2000 and 2014, the estimated reduction in measles deaths has been 86% for the African region, from an estimated 342,000 deaths in 2000 (95% confidence interval between 225,400 – 574,200) to an estimated 48,000 deaths in 2014 (95% confidence interval between 41,600 – 165,000).

This paper documents progress made in the delivery of a wide range of vaccines in the Region using strategies enshrined in the Global Vaccine Action Plan (GVAP) as well as the Regional Immunization Strategic Plan (RISP): 2014-2020.

## Methods

We conducted detailed review of country WHO and UNICEF estimates of immunization coverage data released in July 2015, as the major data source for this paper. We also reviewed programmes reports and records over the period. The analysis employed mainly descriptive statistics to show trends in coverage with the different vaccine antigens in the Region. Tables and figures were also used to illustrate some of the findings in the review.

## Coverage during the period 2013-2015

**Figure 1:**
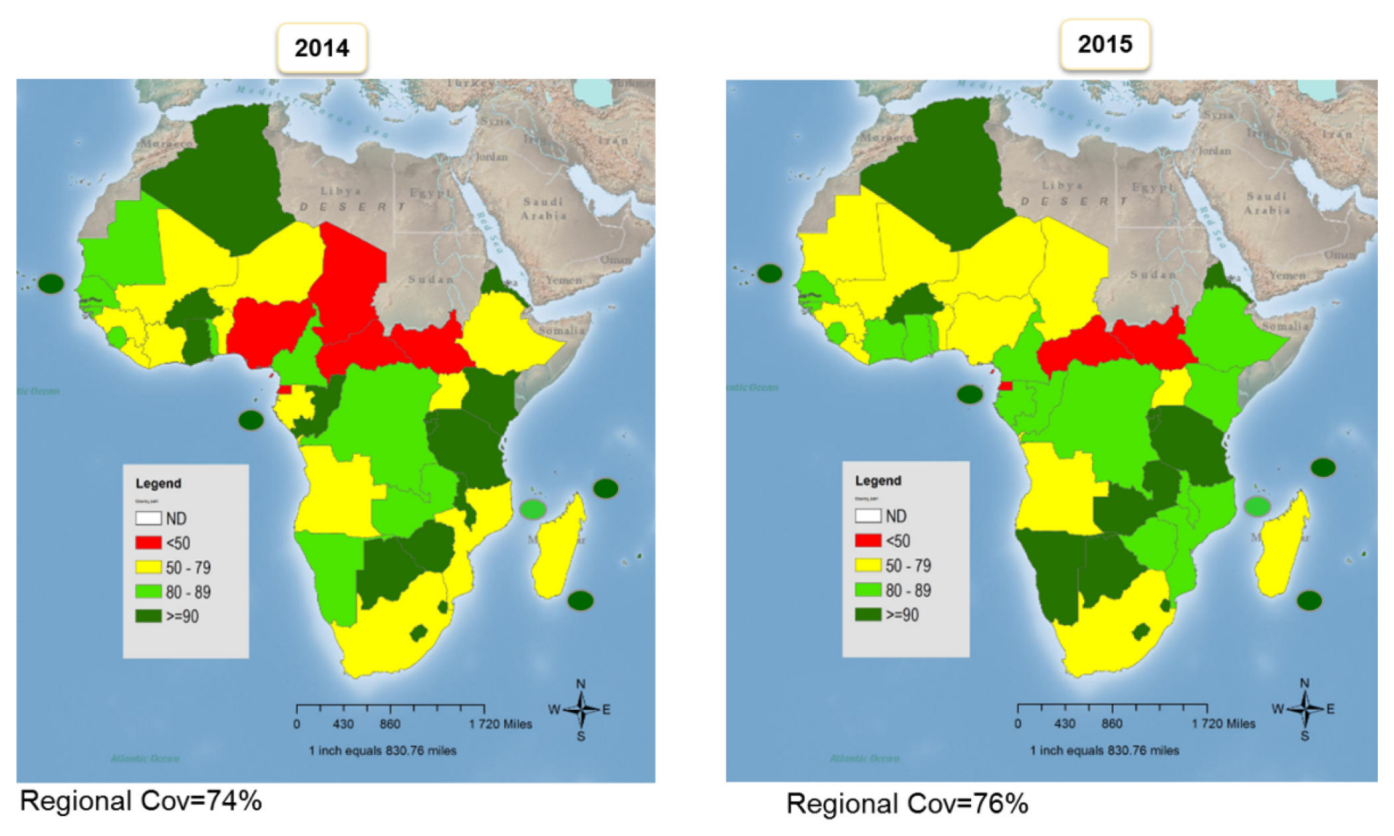
Regional coverage of 3rd dose of Diphtheria Tetanus Pertussis Containing vaccine 2014 and 2015 Source: WHO/UNICEF estimates for 2015, July 2016 release http://apps.who.int/immunization_monitoring/globalsummary/timeseries/tswucoveragedtp3.html

**Figure 2:**
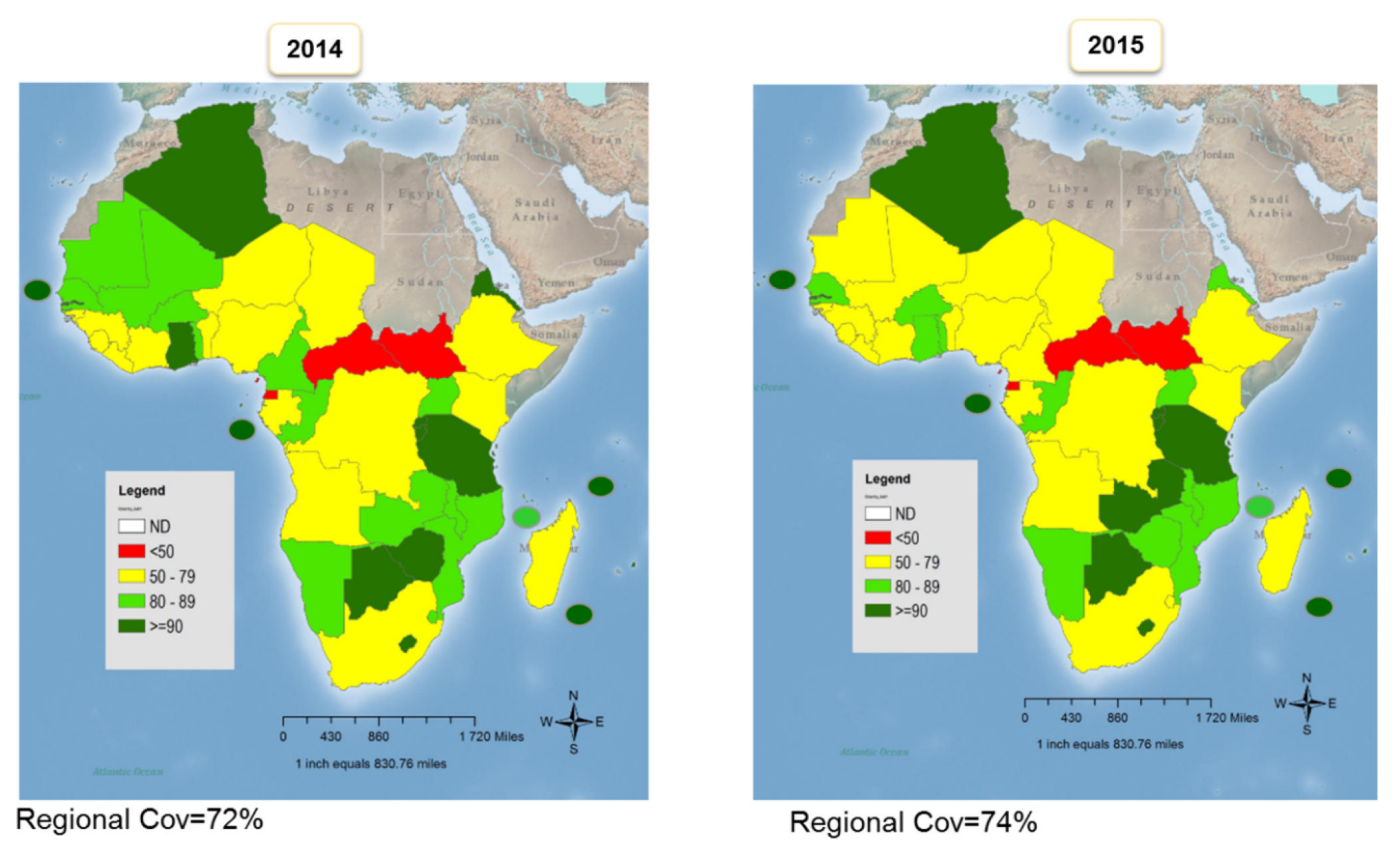
Regional coverage of 1st dose Measles Containing Vaccine (MCV1) 2014-2015.

The 2015 World Health Organization (WHO) and United Nations Children’s Fund (UNICEF) Estimates of National Immunization Coverage (WUENIC) revealed that Regional coverage for DTP3 containing vaccine increased from 76% to 77% between 2014 and 2015. An estimated 24.96 million children were vaccinated with DTP3 in 2015 compared to 23.98 million in 2014. A total of 16 countries reached ≥90% coverage compared to 18 in 2014, and coverage increased in 17 countries including in four of the six priority countries (Chad, Ethiopia, the Democratic Republic of Congo [DRC], and Nigeria). Similarly, two of the countries, Liberia and Sierra Leone, which experienced recent disruption of their public health systems due to Ebola virus outbreak increased their coverage. Other countries are (Benin, Cote d’Ivoire, Eritrea, Ethiopia, Gabon, Gambia, Mozambique, Namibia, Nigeria, Sao Tome, Togo, Tanzania and Zambia (Figure 1 and 2). However, a decrease in the coverage was recorded in 16 countries, including the countries with disruption of their health systems due to persistent internal conflict like South Sudan. Others, such as Madagascar and Kenya, had various challenges including poor implementation of the Reach Every District (RED) approach and effects of recent devolution of governance, respectively. The coverage in Chad, Central Africa Republic (CAR), Equatorial Guinea and South Sudan remained below 50% for both years.

A comparative analysis of the 2015 DTP3 coverage, using administrative data from Countries Joint Reported Form (JRF) and WHO-UNICEF Estimates of National Immunization Coverage (WUENIC), revealed similar coverage in 27 countries. The WUENIC for Botswana, Kenya and Lesotho was higher than the administrative data highlighting possible underreporting from the national administrative system. A comparison of the two datasets in 17 countries revealed gaps between these two sources of data with administrative data reporting higher coverage than what was seen in WUENIC. The highest gap (≥20 points) was observed in Chad, Niger, South Africa, and Mali. Moderate gap (>10 and <20) was observed in South Sudan, Nigeria, Madagascar, DRC, Liberia, Ethiopia, Guinea Bissau and Uganda. Minor gap (≥3 and <10) was observed in Guinea, Zimbabwe, Cote d’Ivoire.

**Figure 3:**
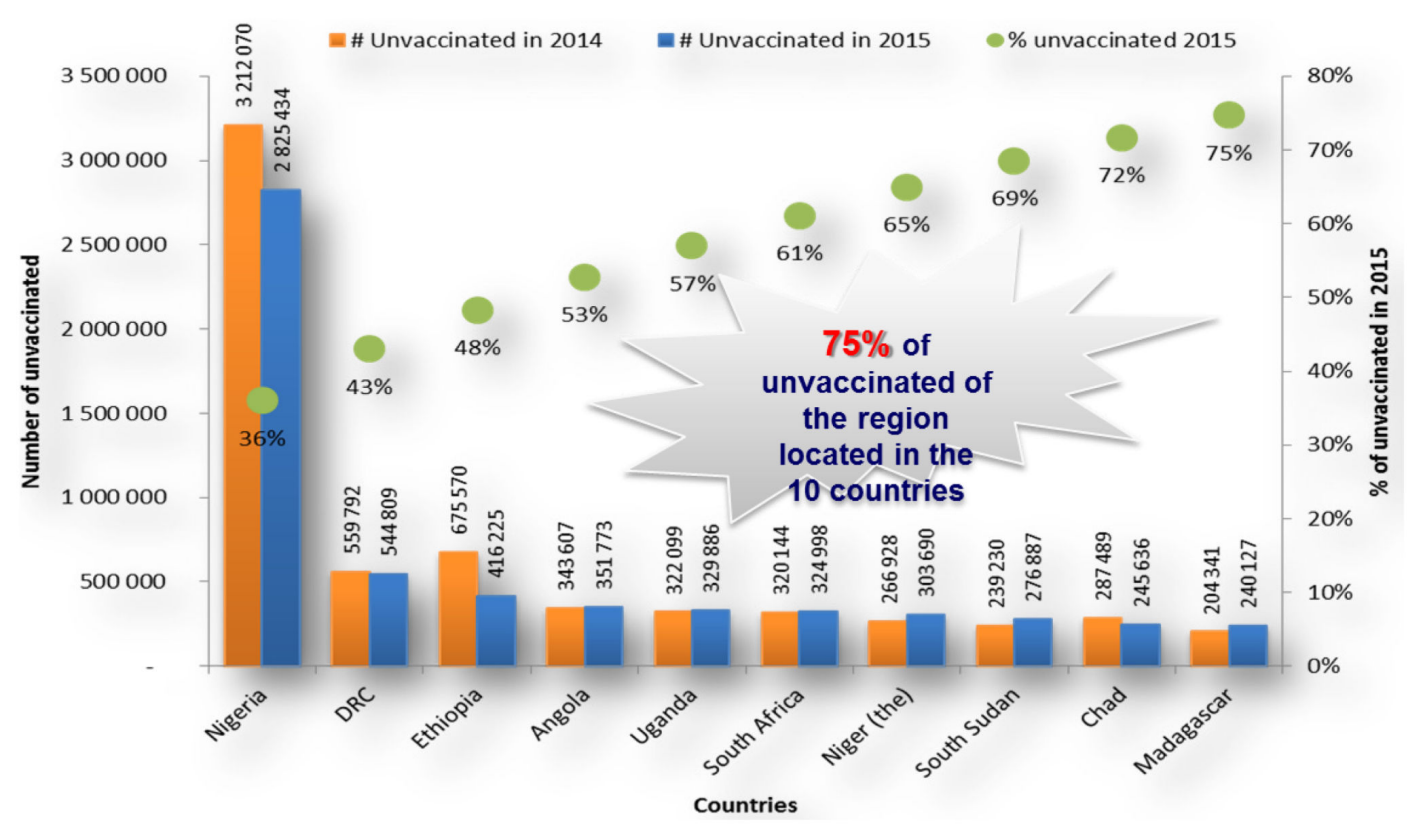
Countries with highest number of DTP3 unimmunized children, 2014-2015

Furthermore, the data revealed that an estimated 7.84 million children were not reached with DTP3 in 2015 compared to 8.2 million in 2014, translating into about 4.3 percentage point reduction in the number of unreached children between the two periods. Approximately 75% of these children are located in 10 countries, with 57% in 5 countries - Nigeria, DRC, Ethiopia, Angola and Uganda as shown in Figure 3. Five of the ten countries with a high number of unreached children are among the priority countries in the African Region, which are being supported in the development and implementation of national immunization coverage improvement plans with specific strategies to reach the unreached children. These are collaborative efforts with other immunization partners.

**Figure 4:**
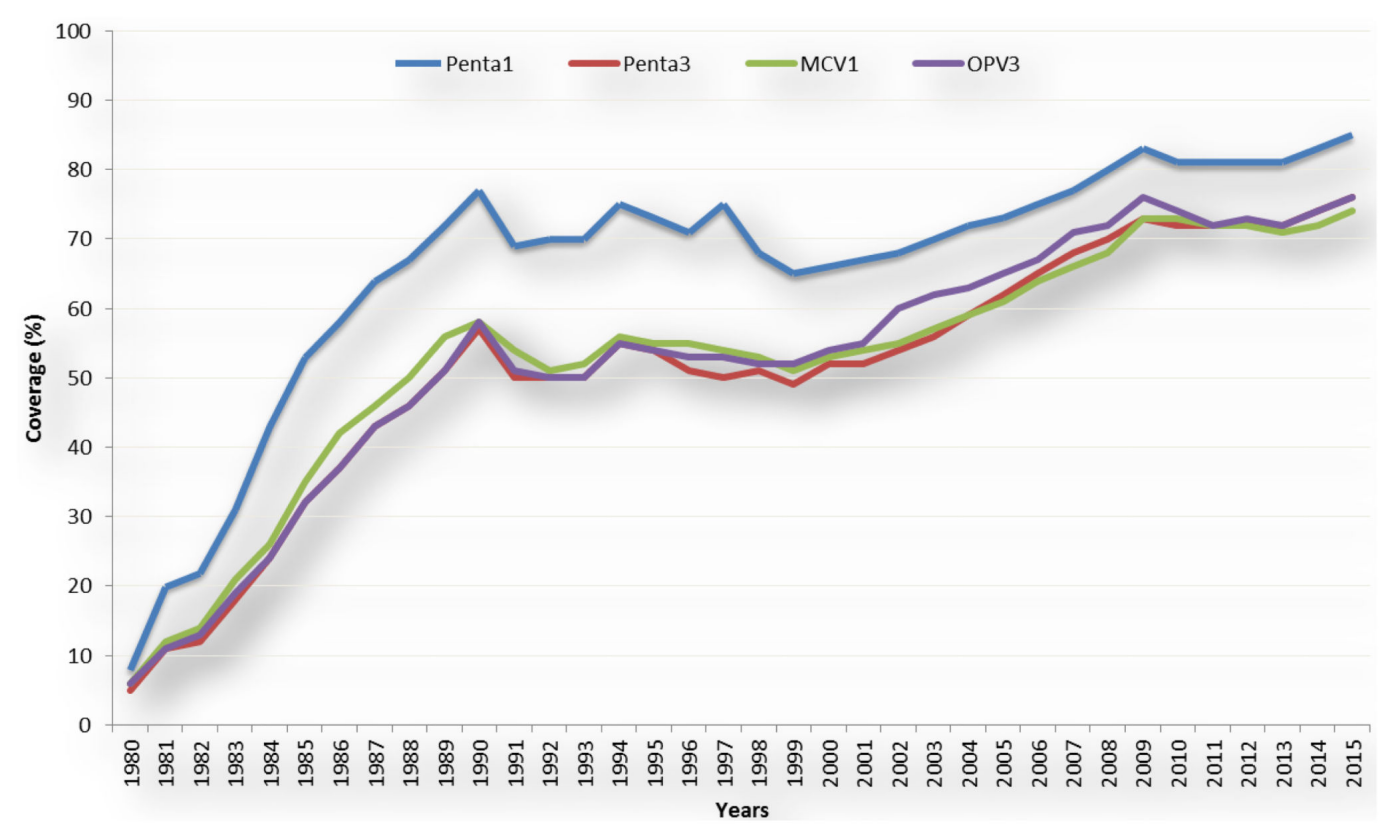
Regional coverage with 1^st^ and 3^rd^ dose of Diphtheria, Tetanus Pertussis containing vaccine (DTP3), 3^rd^ dose of Oral Polio Vaccine ()OPV3, 1^st^ dose of Measles Containing Vaccine, (MCV1) and 1980 - 2015

Similar to the increase in DTP3 coverage, coverage with 1^st^ dose of Measles-Containing Vaccine (MCV1) and 3^rd^ dose of oral polio (OPV3) vaccines have been on a steady rise since 1980 (Figure 4). On immunization against measles, the Region achieved a significant increase in the first dose measles vaccination (MCV1) coverage between 2001 (54% MCV1 coverage WUENIC) and 2009 (73% MCV1 coverage WUENIC). However, Regional MCV1 coverage levels stagnated around 71 – 74% between the years 2009– 2015. This trend is also reflected in the number of countries achieving 90% or more coverage, which increased from only 4 in 2000 to 17 in 2010. In 2015 only 12 countries achieved or maintained MCV1 coverage of 90% or more. Only 12 countries in the Region reached 90% coverage for the first dose of MCV1 while 11 countries sustained 90% coverage during the 2 years. These include Algeria, Botswana, Burundi, Cape Verde, Gambia, Lesotho, Mauritius, Rwanda, Sao Tome et Principe, Seychelles, Tanzania, and Zambia. In 16 countries, MCV1 coverage decreased between 2014 and 2015 with a significant decrease (>5 points) in 7 countries. Three of these countries (CAR, Equatorial Guinea, and South Sudan) reported coverage below 50% for both years.

Moreover, few of the countries with large populations, as well as the countries with challenging infrastructure/programme gaps like Ethiopia, DR Congo, Nigeria, Chad, Madagascar, South Africa, have not been able to improve MCV1 coverage levels in the past 5 years substantially. The MCV1 coverage levels for the years 2011 – 2015 (WUENIC) in the six countries that make up half (53%) of the Region’s population shows that only Tanzania has managed to attain and maintain high coverage.

An analysis of coverage data on all antigens in the Region is shown in Table 1. The Region is seen to be attaining below 90% coverage on all nine antigens reported for 2015. However, unlike in the past, some countries in the Region, such as Algeria and Rwanda have achieved the objective of ≥90% on all antigens delivered in the country. Other countries, such as Burundi, Gambia, Ghana, Mauritius and Sao Tome and Principe, have achieved the objectives of ≥90% national coverage on all but one antigen.

**Figure 5:**
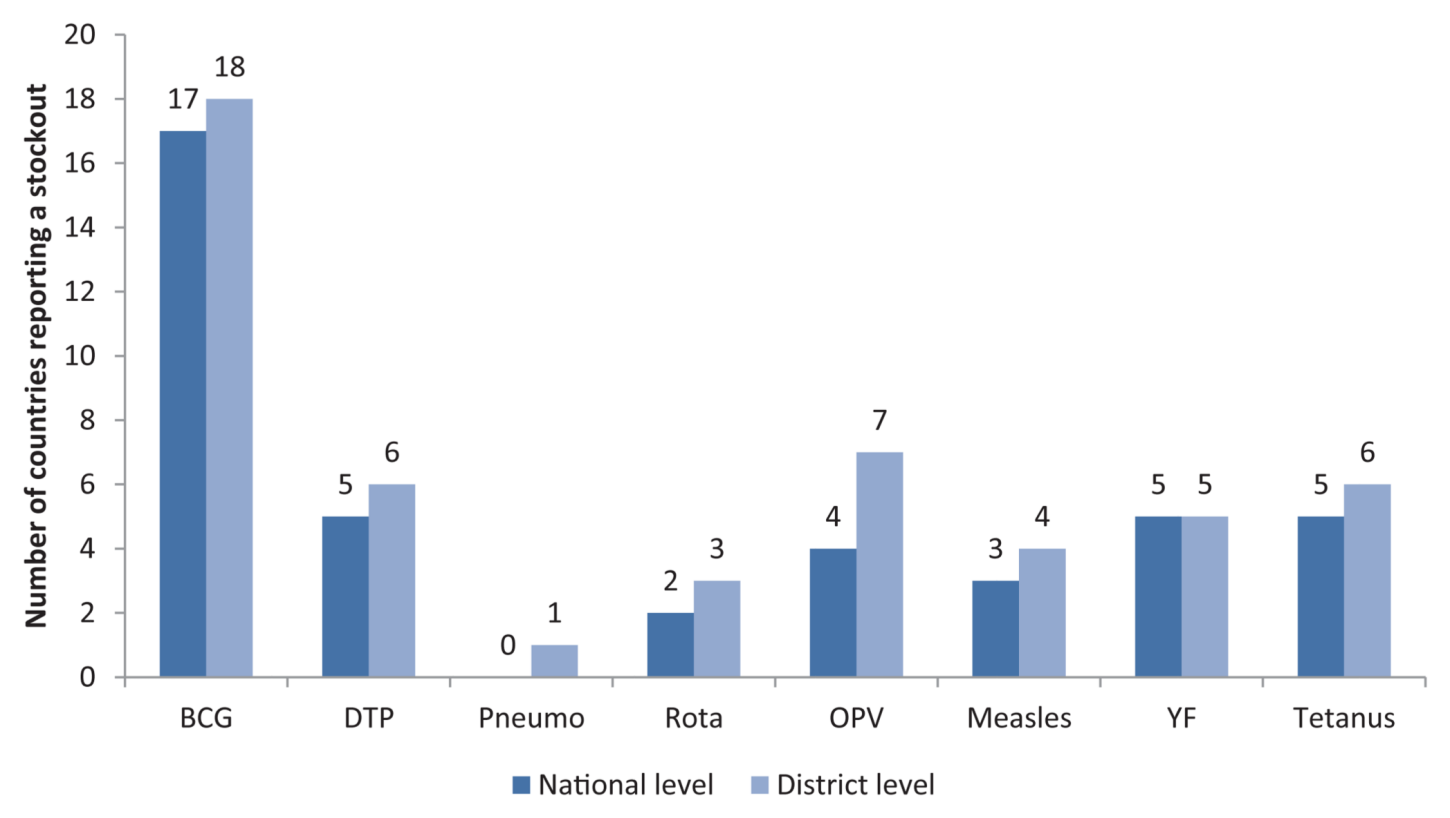
Number of countries that reported vaccine stockouts (in weeks) at the national and district levels in 2015

Failure to meet the vaccination objectives is attributed in part to vaccine stock-outs. Figure 5 shows that in the 2015 JRF, two-fifths of the countries (19/47) in the Region reported at least one episode of stock-out for a duration of at least one week. BCG vaccine has been the most affected antigen for stock-out (19 countries), followed by OPV and DTP-containing vaccine and Tetanus toxoid (6 countries). Most of the stock-outs at district level were due to unavailability of vaccines at the national level. Stock-outs at the district level have probably contributed to the interruption of vaccination at peripheral levels and consequently to the non-achievements of the set objectives in many countries.

Analysis of 2015 JRF data indicates that 43 countries in the region confirmed having a budget line for vaccine procurement. Seven countries reported having funded >90% of their vaccine costs. Thirty countries are funding <50% of the vaccine cost by their national governments.

The situation is completely different for the total funding of routine immunization activities funded by national Governments. Only five countries have funded 100% of their routine immunization costs and 28 countries funded <50%. Eight countries did not provide this information in their 2015 JRF.

## Discussion

The GVAP plans recommend countries to achieve a national coverage ≥ 90% for the third dose of DTP3. The analysis of the 2015 WUENIC data shows that only 13 out of 47 countries (27.6%) have been able to meet the targeted DPT3 coverage for three consecutive years, while 16 out of 47 reached the target in 2015. The surviving infants living in those 13 countries represent only 12% of the total regional target population. The majority of surviving infants in the region still live in countries (34/47) which did not reach the GVAP/RISP recommended target of ≥90% coverage at the national level, which indicates persistent inequities between countries. Five years of the implementation of GVAP 2011-2020 call on more efforts to meet and sustain the momentum towards meeting the set goals. Some countries have however met the GVAP objective on the relevant antigens or were on track for 2015 (see Table 1). Overall, the Region appears to be off track and needs remedial and urgent actions to reach all children with effective vaccine and ensure equity in the coverage.

## Challenges for reaching every child

The challenges facing countries are better appreciated under the different immunization components. For instance, for programme management, monitoring, and accountability, it is observed that there is fragmentation in planning and lack of clear leadership. These factors create gaps in micro-planning and denominator figures (target population issues); gaps in health information and monitoring systems as well as constraints in data quality management, archiving and analysis. There are challenges of ineffective use and interpretation of data to redirect the programme.

Improvements in immunization spending in most African countries have predominantly been due to donor funds. However, of the countries that established line items in their national budgets for routine vaccines, over a third of them did not fund the programmes, and those that had drawn financial plans did not utilize them to the degree expected.

Another challenge is the quality of immunization data in many countries in the Region. Various external evaluations have identified many inconsistencies in reported data suggesting that immunization data monitoring remains weak in most African countries^9^.

Service delivery also suffers a range of shortcomings in the form of insufficient supply and access to quality services, limited service delivery points, and outreach sites. The shortcoming in service delivery is closely related to the inadequate use of the Gavi Alliance health system strengthening funds to strengthen routine immunization activities and is exacerbated with security constraints in some countries of the region^6^.

Another set of challenges has to do with logistic and cold chain issues. Insufficient storage capacity at central and intermediate levels (high proportion of equipment failure or inappropriate) constituted a challenge to the ready availability of vaccines. There also inadequate supply and logistics systems resulting in recurrent shortages or overstock of vaccines and essential commodities as well as lack of funding for vaccine distribution at the most peripheral levels. Cold chain management in resource-poor settings, where electricity is non-existent or erratic, coupled with a lack of adequate trained staff to administer vaccines present major challenges in most African countries. Furthermore, of those children who do receive the vaccines, some receive them late or at inappropriate timing, and likely receive sub-optimal disease protection.

Furthermore, weak communication strategies at all levels; insufficient demand creation, weak links with communities and their leaders and low community awareness and participation have all been identified as impediments to attaining the GVAP objectives. The GVAP recognized these and highlighted the need to promote community participation to engender demand for immunization services. In one of its targets, it was stated that individuals and communities should understand the values of vaccines and demand same as right and recognize their responsibilities too. Unfortunately, this seems to be lacking. The principle of country ownership also needs to be promoted. The analysis here reveals that very few countries provide funding for immunization.

## Recommendations

As we pursue the targets of the Sustainable Development Goals (SDGs), the Region is cognizant of the various constraints and challenges to reaching every child. Since assuming office as the Regional Director, Dr. Moeti, has taken steps to ensure that these challenges are addressed and that the goal of equity in access to health is achieved in the Region. The Ministerial Conference held in February 2016 in Addis Ababa provided an opportunity to assess immunization programmes in Africa and the role of the government critically, take ownership of the Regional and country-specific problems, and develop precise strategies to overcome the challenges identified. The underlying theme for this ministerial event was to foster country ownership for sustainable funding to achieve the GVAP goals and targets while advocating for greater engagement with communities, civil society organizations and other partners for sustainable demand for immunization.

One of the key gaps recognized at the conference is the need to commit government resources for sustainable high and equitable immunization coverage. Thus the conference culminated in the signing of the Addis Declaration on Immunization (ADI) by ministers across Africa, committing them to 10 specific goals to achieve universal access to immunization in Africa. There is a need for increased immunization systems strengthening, as many are plagued by weak infrastructure and shortage of skilled human resources. More affordable and adapted vaccines need to be made available. Other key actions include strengthening the integration with other child survival and high impact interventions and extend the benefits of immunization to adolescents and adults.

The new Regional Strategic Plan for Immunization 2014-2020 which is intended to provide policy and programmatic guidance to Member States to optimize immunization services, in line with the GVAP, will be used to strengthen their immunization programmes further. Key approaches in the Regional Strategic Plan include integrating immunization into national health policy and plan and during emergencies, strengthening immunization financing, enhancing partnerships, building national capacity, improving monitoring and data quality, improving vaccine management, safety and regulation and promoting implementation, research, and innovations.

In conclusion, while commending countries in the African Region for giant steps made in EPI performance over the past four decades, there exist wide inter- and intra-country differences, with a significant number of children remaining unreached, un/under-vaccinated, and still dying from VPDs. Immunization systems’ strengthening is essential in the framework of the overall health system strengthening, as most health facilities are under-staffed with inadequate resources to function efficiently. Issues of vaccine supply, financing, and sustainability require urgent attention. Increased political and financial commitment from governments as well as coordinated national evidence-informed efforts by all immunization stakeholders are needed to both maintain current achievements and make additional progress for EPI in the African Region. African leaders must be held accountable for meeting agreed country targets and honouring international commitments they have signed on to.

## Figures and Tables

**Table 1:** Regional coverage on all antigens by country for 2015

Country	BCG		Penta 1		Penta 3		MCV1		MCV2		PAB		PCV3		OPV3		RCV1		Rota Last		YFV	
	2015	2014	2015	2014	2015	2014	2015	2014	2015	2014	2015	2014	2015	2014	2015	2014	2015	2014	2015	2014	2015	2014
Algeria	99	99	99	99	95	95	75	68	99	99	92	92			95	95						
Angola	79	81	77	81	64	64	97	97	26		78	78	58	45	70	68			49	18	72	77
Benin	89	93	90	90	79	75	88	88			85	93	74	70	79	75					71	64
Botswana	98	98	98	98	95	95	93	94	85	85	92	92	81	81	96	96			82	82		
Burkina Faso	98	98	95	95	91	91	92	93	50	17	92	89	91	91	91	91	68		91	91	88	88
Burundi	93	92	97	98	94	95	79	80	65	60	85	85	94	95	94	95			96	96		
Cabo Verde	94	99	97	99	93	95	49	49	95	79	92	92			93	95	95	79				
Cameroon	74	82	92	93	84	87	62	54			85	85	85	87	83	86			73	46	77	80
Central African Republic (the)	74	74	69	69	47	47	81	80			60	60	47	47	47	47					48	48
Chad	70	59	60	60	55	46	80	80			75	60			62	54					49	40
Comoros (the)	73	76	81	83	80	80	72	62			85	85			81	79						
Congo (the)	85	95	85	95	80	90	79	77			85	85	80	85	80	90			80	60	65	65
Côte d'Ivoire	79	84	99	93	83	76	27	44			85	82	72		81	76					49	49
Democratic Republic of the Congo (the)	74	78	82	81	81	80	85	90			82	82	73	61	78	79					65	65
Equatorial Guinea	48	56	28	59	16	20	78	70			70	70			17	24						
Eritrea	97	97	98	97	95	94	68	61	75		94	94			95	94			96	25		
Ethiopia	75	75	94	86	86	77	97	96			80	80	85	76	85	75			83	63		
Gabon	98	91	87	77	80	70	89	92			85	85			79	68					68	60
Gambia (the)	98	96	99	98	97	96	52	52	77	73	92	92	97	96	96	97			97	92	97	96
Ghana	97	99	97	99	88	98	69	69	63	67	88	88	88	93	88	93	89	92	88	98	88	92
Guinea	72	72	60	60	51	51	75	79			80	80			42	42					53	53
Guinea-Bissau	94	94	92	92	80	80	90	90			80	80	10		78	78					69	53
Kenya	87	94	96	97	89	92	64	58	28		80	76	75	81	83	93			66	19	1	1
Lesotho	98	98	98	98	93	93	58	64	82	82	83	83	29		90	90						
Liberia	74	73	77	74	52	50	87	85			89	89	56	45	52	49					56	54
Madagascar	70	75	79	83	69	73	76	80			78	78	69	72	71	73			69	39		
Malawi	90	97	93	97	88	91	95	95	8		89	89	88	87	88	87			84	83		
Mali	79	79	80	90	68	77	55	60			85	85	58	78	76	84			33	13	64	64
Mauritania	85	98	87	88	73	84	70	84			80	80	71	71	67	84			56	5		
Mauritius	98	97	98	97	97	97	99	98	85	85	95	95			98	98	99	98	66			
Mozambique	95	94	90	92	80	79	85	85			83	83	80	73	80	79			17			
Namibia	94	97	98	92	92	88	85	83			85	85	81		92	88			87			
Niger (the)	77	76	85	89	65	68	73	72	16	3	81	81	74	13	65	67			70	19	72	70
Nigeria	68	64	70	64	56	49	54	51			55	55	13		55	49					54	51
Rwanda	99	99	99	99	98	98	97	97	87		90	90	98	98	98	98	97		98	98		
Sao Tome and Principe	97	95	98	98	96	95	93	92	76	71	99	99	96	95	96	95					93	92
Senegal	95	95	94	94	89	89	80	80	54	13	91	91	89	81	85	85	80	80	83		80	80
Seychelles	99	98	99	99	97	99	98	99	98	98	99	99			97	99	98	99				
Sierra Leone	90	90	95	88	86	83	76	78	60		85	85	86	83	86	83			85	53	78	80
South Africa	69	77	72	73	69	70	76	70	63	60	80	80	69	65	70	71			72	72		
South Sudan	43	46	49	49	31	39	20	22			68	64			41	44						
Swaziland	98	99	96	99	90	98	78	86	89	89	88	88	88	67	98	98			36			
Togo	86	79	92	91	88	87	85	82			81	81	86	34	88	85			85	35	85	82
Uganda	93	93	89	89	78	78	82	82			85	85	66	50	82	82						
United Republic of Tanzania	99	99	99	99	98	97	99	99	57	29	90	88	95	93	96	97	99	99	98	97		
Zambia	99	99	97	96	90	86	90	85	47	33	85	85	86	77	90	78			82	73		
Zimbabwe	90	99	94	98	87	91	86	92			75	75	87	91	88	92			87	48		
